# Allogeneic CAR-T cells with of HLA-A/B and TRAC disruption exhibit promising antitumor capacity against B cell malignancies

**DOI:** 10.1007/s00262-023-03586-1

**Published:** 2024-01-17

**Authors:** Xinfeng Chen, Binghe Tan, Haizhou Xing, Xuan Zhao, Yu Ping, Zhen Zhang, Jianmin Huang, Xiujuan Shi, Na Zhang, Boxu Lin, Weijie Cao, Xin Li, Xudong Zhang, Ling Li, Zhongxing Jiang, Mingzhi Zhang, Wei Li, Mingyao Liu, Bing Du, Yi Zhang

**Affiliations:** 1 Biotherapy Center, The First Affiliated Hospital of Zhengzhou University, Zhengzhou, 450052 Henan China; 2 Cancer Center, The First Affiliated Hospital of Zhengzhou University, Zhengzhou, 450052 Henan China; 3 Shanghai Frontiers Science Center of Genome Editing and Cell Therapy, Shanghai Key Laboratory of Regulatory Biology, Institute of Biomedical Sciences and School of Life Sciences, East China Normal University, Shanghai, 200241 China; 4 BRL Medicine Inc, Shanghai, 201109 China; 5 Department of Hematology, The First Affiliated Hospital of Zhengzhou University, Zhengzhou, 450052 Henan China; 6 State Key Laboratory of Esophageal Cancer Prevention & Treatment, Zhengzhou, 450052 Henan China; 7 School of Life Sciences, Zhengzhou University, Zhengzhou, 450052 Henan China; 8 Engineering Key Laboratory for Cell Therapy of Henan Province, Zhengzhou, 450052 Henan China

**Keywords:** Universal CAR-T cell, CRISPR/Cas9, NK cell, HLA, B cell malignancies

## Abstract

**Background:**

Although chimeric antigen receptor T (CAR-T) cells have been proven to be an effective way of treating B cell malignancies, a lot of patients could not benefit from it because of failure in CAR-T cell manufacturing, disease progression, and unaffordable price. The study aimed to explore universal CAR-T cell products to extend the clinical accessibility.

**Methods:**

The antitumor activity of CRISPR/Cas9-edited allogeneic anti-CD19 CAR-T (CAR-T19) cells was assessed in vitro, in animal models, and in patients with relapsed/refractory (R/R) acute B cell lymphoblastic leukemia (B-ALL) or diffuse large B cell lymphoma.

**Results:**

*B2M*^*−*^*/TRAC*^*−*^ universal CAR-T19 (U-CAR-T19) cells exhibited powerful anti-leukemia abilities both in vitro and in animal models, as did primary CD19^+^ leukemia cells from leukemia patients. However, expansion, antitumor efficacy, or graft-versus-host-disease (GvHD) was not observed in six patients with R/R B cell malignancies after U-CAR-T19 cell infusion. Accordingly, significant activation of natural killer (NK) cells by U-CAR-T19 cells was proven both clinically and in vitro. *HLA-A*^*−*^*/B*^*−*^*/TRAC*^*−*^ novel CAR-T19 (nU-CAR-T19) cells were constructed with similar tumoricidal capacity but resistance to NK cells in vitro. Surprisingly, robust expansion of nU-CAR-T19 cells, along with rapid eradication of CD19^+^ abnormal B cells, was observed in the peripheral blood and bone marrow of another three patients with R/R B-ALL. The patients achieved complete remission with no detectable minimal residual disease 14 days after the infusion of nU-CAR-T19 cells. Two of the three patients had grade 2 cytokine release syndrome, which were managed using an IL-6 receptor blocker. Most importantly, GvHD was not observed in any patient, suggesting the safety of *TRAC*-disrupted CAR-T cells generated using the CRISPR/Cas9 method for clinical application.

**Conclusions:**

The nU-CAR-T19 cells showed a strong response in R/R B-ALL. nU-CAR-T19 cells have the potential to be a promising new approach for treating R/R B cell malignancies.

**Supplementary Information:**

The online version contains supplementary material available at 10.1007/s00262-023-03586-1.

## Summary

Exploring universal CAR-T19 cells (U-CAR-T19) through gene-edited methods (TALEN or CRISPR/Cas9) is strategy to resolve the limitation of autologous CAR-T19 cells in clinic, such as failure, time-consuming, and expansive cost of CAR-T cell preparation. However, (*TRAC*^*−*^*/CD52*^*−*^) U-CAR-T19 cells exhibited antitumor ability in B-ALL in clinic depending on anti-CD52 antibody. Herein, we initially explored a new method to manufacture (*TRAC*^*−*^*/B2 M*^*−*^) U-CAR-T19 cells with satisfied antitumor response in preclinical models while inefficiency in clinic. NK cell activation and killing mediated the rejection of U-CAR-T19 cells were the reasons for the unsatisfied clinical results. Therefore, we further manufactured nU-CAR-T19 (*HLA-A*^*−*^*/B*^*−*^*/TRAC*^*−*^) cells, which have been verified as a promising new approach for treating R/R B cell malignancies, and provided a novel strategy for the development of U-CAR-T cells.

## Background

Autologous anti-CD19 chimeric antigen receptor T (CAR-T19) cell drugs have been approved to treat relapsed or refractory (R/R) B cell acute lymphoblastic leukemia (B-ALL) in patients up to 25 years old in the USA and Europe [1]. In addition, an increasing number of CAR-T19 cell products have been approved for the treatment of R/R specific B cell lymphoma [2–5]. However, the manufacturing of autologous CAR-T cells is expensive and time-consuming, which limits their scale and industrialization. Furthermore, most patients would not benefit from autologous CAR-T19 cells because of the failure of CAR-T cell preparation or rapid disease progression [6]. Thus, exploring allogeneic CAR-T cells, an “off-the-shelf” CAR-T cell product using multiple gene-edited methods for immediate clinical application, has become the focus in the field of cancer immunotherapy [7, 8]. CRIPSR/Cas9 technology has emerged as a simple and efficient method to modify target cell genes [9, 10]. Meanwhile, it was reported that CRISPR/Cas9 editing T cells is feasible for cancer immunotherapy [11]. To prevent graft-versus-host-disease (GvHD) or rejection of HLA-incompatible cells, the T cell receptor alpha constant (*TRAC*) and *B2M* (necessary for HLA class I assembly and presentation) were edited using CRISPR/Cas9 technology in donor-derived allogeneic CAR-T19 cells and named universal CAR-T19 (U-CAR-T19) cells [12]. The antitumor capacities of several allogeneic CAR-T cell products manufactured using different strategies have been confirmed in clinical [13–15]. In the present study, the antitumor capacities of U-CAR-T19 cells (*TRAC*^*−*^*/B2M*^*−*^) edited using CRISPR/Cas9 were confirmed to be equivalent to that of normal CAR-T19 cells, both in vitro and in an animal model. Next, a clinical trial was conducted to verify the safety and efficacy of an "off-the-shelf" allogeneic CAR-T cell product targeting CD19 in patients with R/R B cell malignancies. Here, six patients with R/R B cell malignancies who treated with gene-edited allogeneic U-CAR-T19 cells were reported, and natural killer (NK) cell activation was found to limit their persistence, expansion, and antitumor ability. HLA-I molecules, particularly HLA-C/E, mediate resistance of NK cell killing [16]. Thus, by altering the gene-editing strategy, *HLA-A*^*−*^*/HLA-B*^*−*^*/TRAC*^*−*^ novel U-CAR-T19 (nU-CAR-T19) cells were produced and used to treat another three patients with R/R B-ALL. All the three patients achieved a minimal residual disease (MRD)-negative remission, which suggests the significant potential of nU-CAR-T19 cells in treating B cell malignancies.

## Methods

### Characteristics of gene-edited allogeneic CAR-T19 cells

**Fig. 1:**
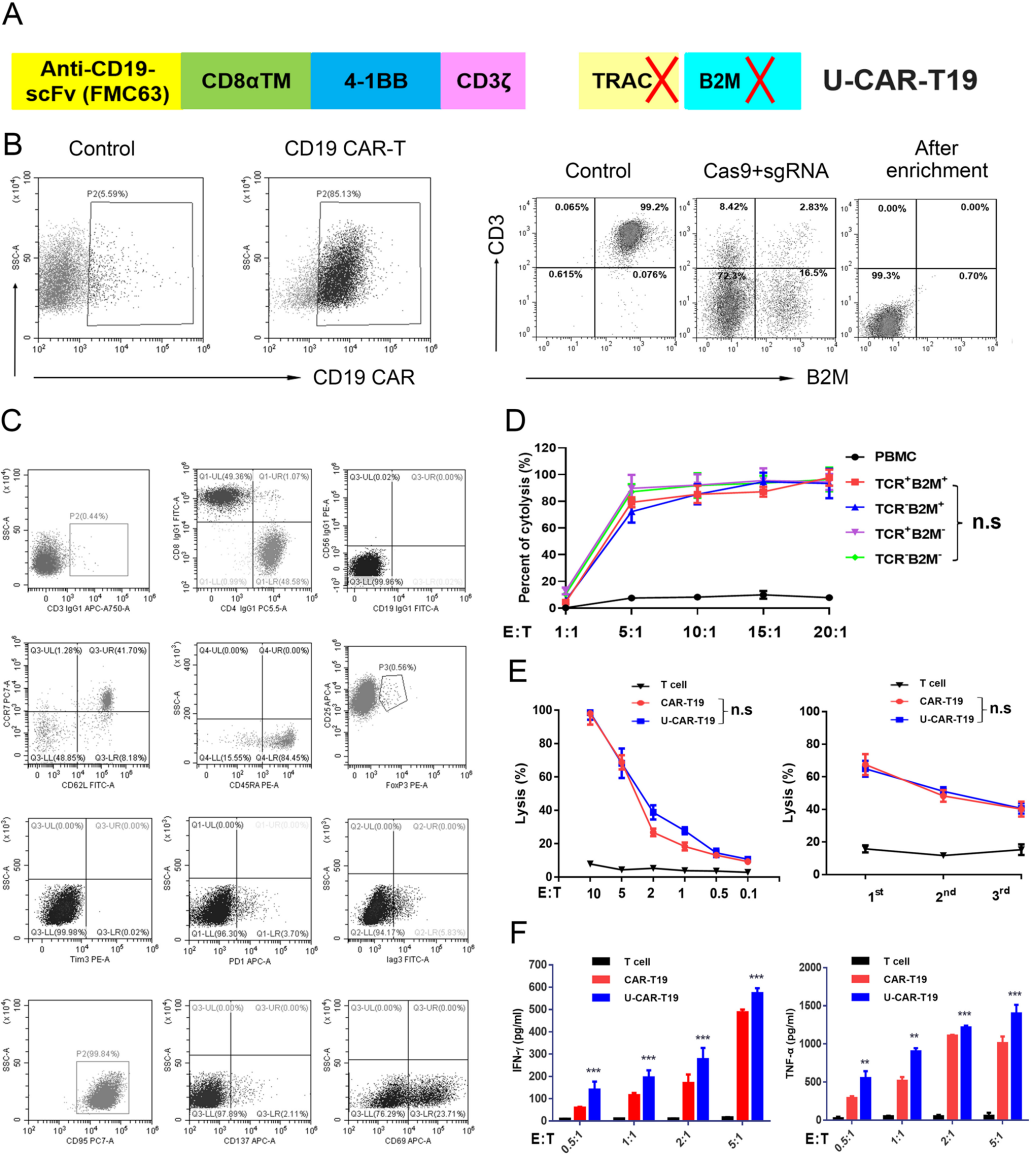
Construction of *TRAC*^*−*^*/B2M*^*−*^ allogeneic CAR-T19 cells using the CRISPR/Cas9 method. **a** The second-generation anti-CD19 CARs were constructed with antibody clone FMC63, hinge and transmembrane regions from CD8, 4-1BB (CD137), and CD3ζ. Then, *TRAC* and *B2M* were knockout by CRISPR/Cas9 to get U-CAR-T19 cells. **b** Representative images of CAR-T19 and U-CAR-T19 cells. CAR-T19 cells were electroporated with RNP comprising of Cas9 and sgRNAs that target *TRAC* and *B2M* loci and cultured for 7 days. Both membrane CD3 complex and B2M expression were detected by flow cytometry following enrichment and purification with magnetic beads to remove CD3^+^ CAR-T cells. **c** Expression of markers associated with T cell activation, exhaustion, memorization, and regulatory (CD56, CCR7, CD62L, CD45RA, CD25, FoxP3, Tim-3, PD-1, LAG3, CD95, CD137, and CD69) were detected by flow cytometry. **d** *TCR* and/or *B2M*-deficient cells were stimulated with Raji cells at the given effector/target (E/T) ratios. The 24 h cytolysis of effector cells to target cells was detected by measuring the release of LDH. **e** Specific lysis of U-CAR-T19 cells at different E/T ratios and different rounds of stimulation with target cells (1:1 for 24 h). CAR-T19 and U-CAR-T19 cells were co-cultured with Raji cells to perform the cytolysis assay, and the E/T ratios and stimulation are shown in the figure. The cytolysis of effector cells to target cells was detected by measuring the release of LDH. (F) IFN-γ and TNF-α expressions were determined by ELISA after co-culture of CAR-T19/U-CAR-T19 and target cells (18 h). Statistical significance, ANOVA with Tukey’s multiple comparison test or two-tailed, paired Student’s *t* test were used, n.s, no significance, *P < 0.05, **P < 0.01, ***P < 0.001

Second-generation anti-CD19 CARs were constructed using single-chain antibody fragments derived from antibody clone FMC63, hinge and transmembrane regions from CD8, intracellular domain from 4-1BB (CD137), and intracellular domain from CD3ζ. Anti-CD19 CARs were cloned into the pCDH-EF1α backbone, downstream of the EF1α promoter (Fig. 1a). The lentivirus was produced by transfecting 293 T cells with CAR plasmids, pMD2.G, and psPAX2, using polyethylenimine. Viral supernatants were harvested after 3 days to transduce primary human T cells stimulated for 2–3 days. CAR-T19 cells were generated by transducing anti-CD3/CD28 bead-activated human primary T cells purified using CD8 and CD4 beads from peripheral blood mononuclear cells (PBMCs) of health donors with concentrated lentivirus vectors at an MOI of 10.

### Gene knockout in CAR-T19 cells

For gene knockout, Cas9 and sgRNAs targeting *TRAC*, and *B2M*, or *HLA-A/B* were transfected into T cells by electroporation. In brief, single 10^7^ T cells were electroporated with 25 μg spCas9 and 10 μg sgRNAs in the indicated buffer to a total volume of 100 μL. On day 9 after stimulation, CD3^−^ T cells were sorted by microbead depletion. CAR positive cells in CAR-T cell products were not purified but met quality control criteria when analyzed by flow cytometry. The results showed that the rate of CD3 negativity in purified cells was > 99%, and the rates of HLA-A, HLA-B, and B2M negativity were > 70% in allogeneic CAR-T19 cells. All CAR-T cell products had CAR positive rates of over 50%. CAR copy numbers of each CAR-T cell product were detected by qPCR and with a range of 2.02 ~ 2.68 copies/cell. The sgRNAs used were as follows: *TRAC* sgRNA (5′-AGAGCAACAGTGCTGTGGCC-3′, chr14:22,547,693, exon 1 of *TRAC*), *B2M* sgRNA (5′-GAGTAGCGCGAGCACAGCTA-3′, chr15:44,711,569, exon 1 of *B2M*), *HLA-A* sgRNA (5′-GUAUGGCUGCGACGUGGGGU-3′, chr6:29,942,532, exon 3 of *HLA-A*), *HLA-B* sgRNA (5′- GUACGGCUGCGACGUGGGGC-3′, chr6:3,353,875, exon 3 of *HLA-B*). The antitumor abilities of (*n*)U-CAR-T19 cells in vitro and in vivo were evaluated. NK cell activation-mediated rejection of U-CAR-T19 cells was explored as described.

### Cells

PBMCs from seven healthy donors were isolated by density gradient centrifugation using the Ficoll reagent (Sigma-Aldrich, MO, USA). T cells were purified via magnetic separation using anti-CD8/CD4 microbeads (Miltenyi Biotech, Bergisch Gladbach, Germany) and activated using T Cell TransAct (Miltenyi Biotech). T cells were cultured in X-VIVOTM 15 medium (Lonza, Basel, Switzerland) supplemented with 2% human AB serum or CTS™ Immune Cell Serum Replacement (ThermoFisher, MA, USA) and recombinant human IL-2 (100 units/mL), IL-7 (5 ng/mL) and IL-15 (5 ng/mL). Cells were harvested once the number reached the requirement for administration, and then washed, formulated, and cryopreserved. The 293 T and Raji cells were purchased from the Cell Bank of the Chinese Academy of Sciences. 293 T cells were maintained in DMEM (Gibco, NY, USA) supplemented with 10% fetal bovine serum (FBS) (ThermoFisher). Raji cells were cultured in RPMI1640 medium (Corning, NY, USA) supplemented with 10% FBS. Raji cells stably expressing firefly luciferase (fLuc) were established by lentiviral infection. All the stable cell lines were selected using puromycin.

### Flow cytometry

CAR and membrane protein expression levels were determined using flow cytometry. Cells were pre-washed and incubated with antibodies for 30 min on ice. After two washes, the samples were run on an LSR Fortessa (BD Biosciences, NJ, USA) and analyzed using Flow Jo software. The following antibodies were used, anti-human CD3, CD4, CD45RA, CD62L, CCR7, CD25, CD56, CD69, CD95, CD95, LAG3, Tim-3, PD-1, FOXP3, CD137, B2M, HLA-A, HLA-B, and carboxyfluorescein diacetate succinimidyl ester (CFSE) (BD Biosciences). To detect CAR expression, biotinylated human CD19 (amino acids 20–291), protein (ACRO Biosystems, DE, USA), and PE Streptavidin (BioLegend, CA, USA) were added sequentially.

#### ELISA

For in vitro evaluation of activation-related cytokines, CAR-T cells were co-cultured with Raji cells at an effector/target (E/T) ratio of 1:1 in media without exogenous cytokines. The supernatant was collected after 18–24 h, and IFN-γ and TNF-α secretion were measured using human IFN-γ/TNF-α ELISA Kits (Invitrogen, MA, USA) according to the manufacturers’ instructions.

### Cytotoxicity assay

Wild-type (WT) T cells or CAR-T cells were co-cultured with Raji cells at the indicated E/T ratios. Cytotoxicity was measured by the release of lactate dehydrogenase (LDH) using the CytoTox 96® Non-Radioactive Cytotoxicity Assay (Promega, WI, USA), according to the manufacturer’s instructions. These experiments were repeated three times.

### Human primary NK cell isolation and culture

Human primary NK cells were isolated using a negative selection kit (RosetteSep™ Human NK Cell Enrichment Cocktail; Stem Cell Technologies, Vancouver, Canada). The isolated NK cells were cultured in RPMI 1640 with l-glutamine (Corning), supplemented with 10% FBS, 1% penicillin/streptomycin and 1000 IU/mL recombinant human IL-2. For NK cell co-culture, resting NK cells were isolated from fresh PBMCs using CD56 Micro Beads enrichment and MS columns (Miltenyi Biotech). In co-culture experiments with NK cells, allogeneic CAR-T cells and freshly isolated resting NK cells were mixed in a 1:1 cellular ratio. The remaining target cells were quantified using flow cytometry.

### CFSE-mixed lymphocyte reaction (MLR) assay

The CFSE-MLR assay was performed to monitor allogeneic immune cell-mediated rejection or killing. For assays evaluating allogeneic rejection, nU-CAR-T19 and WT T or U-CAR-T19 cells were termed as target cells, and allogeneic PBMCs, T cells, or NK cells were used as effector cells. Briefly, target cells were irradiated with X-rays (30 Gy) and labeled with CFSE. Both the target and effector cells were adjusted to 1 × 10^6^ cells/mL in RPMI medium (Corning) and co-cultured in a total volume of 1 mL of medium in 48-well flat-bottom plates at 37 °C in a 5% CO_2_ incubator in the dark for the indicated days. After MLR culture, the cells were harvested and analyzed using flow cytometry. The remaining CFSE-positive cells represented the target cells that were not killed by effector cells. The ratio of residual CFSE-positive cells divided by the initial number of CFSE-positive cells was donated as Lysis %.

### *In vivo* mouse experiments

All animal experiments were conducted in compliance with the “*Guide for the Care and Use of Laboratory Animals* (2011)” issued by the National Research Council (USA), “*Laboratory Animal Administration Regulation* (2017)” issued by the National Science and Technology Committee (China), and the Laboratory Animal Administration regulations (Shanghai, Jiangsu). Animal care and use was reviewed and approved by the Institutional Animal Care and Use Committee of the test facility. For the experiments, 6–8-week-old male B-NDG (NOD.CB17-PrkdcscidIl2rgtm1/Bcgen) mice were intravenously injected with 2 × 10^5^ ffLuc-transduced Raji cells. CAR-T cells (2 × 10^6^) in each group were administered intravenously after 5 days. Bioluminescent images were acquired and analyzed using an IVIS imaging system and software (PerkinElmer, MA, USA). Survival, serum cytokine concentration, and body weight of mice in each group were recorded at the indicated time points. The experiments were performed at the East China Normal University Center for Animal Research.

### GvHD studies

For in vivo GvHD studies, T cells (2 × 10^6^ cells/mouse) were resuspended in PBS and infused intravenously into sublethal X-ray irradiated (9 Gy/mouse) NOD mice. The mice were monitored for GvHD every 2 days. The following signs were included in the clinical indices: serum IFN-γ level, weight loss, hunching, activity, fur texture, and skin integrity. The moribund mice were euthanized for ethical reasons.

### Clinical trial study design and participants

This ongoing phase 1 clinical trial is evaluating the safety and tolerability of (*n*)U-CAR-T19 cells in patients with R/R B-ALL or B cell lymphoma (NCT03229876). Written informed consents were obtained from all the patients or their families before initiating treatment. Six and three patients with R/R B-ALL (16–35 years old) and B cell lymphoma (18–65 years old), respectively, were enrolled and received (*n*)U-CAR-T cell therapy. The major enrollment criteria included the presence of > 5% CD19-positive leukemic blasts in the bone marrow of patients with B-ALL and measurable lesions of lymphoma, without the chance of receiving autologous CAR-T19 cell therapy. The key exclusion criteria included active infection, allogeneic hematopoietic stem cell transplantation, and active tumor invasion of the nervous system.

### Procedures

After inclusion, patients started 6 days of lympho-depletion treatment before allogeneic CAR-T19 cell (manufactured as described above) infusion on day 0. All patients then entered a follow-up period of 3–12 months. The lympho-depletion regimen comprised fludarabine (25 mg/m^2^, D-6 to D-4), cyclophosphamide (500 mg/m^2^, D-6 to D-5), with or without etoposide (100 mg/day, D-5 to D-4). In the current study, all patients received allogeneic CAR-T19 cells at a dose of 1–6 × 10^6^/kg splitting into two injections. Following cell infusion, the toxicities associated with lympho-depletion or allogeneic CAR-T19 cells were monitored and recorded. Disease response was assessed weekly for 28 days after allogeneic CAR-T19 cell infusion using flow cytometry to evaluate MRD. PET-CT was used to evaluate the curative effect in treated lymphoma patients at 1 or 3 months after allogeneic CAR-T19 cell infusion. Cytokine analysis was used to evaluate CAR-T cell-associated cytokine release syndrome (CRS), and qPCR was used to detect the presence and expansion of (n)U-CAR-T19 cells in vivo*.*

### Outcome

The primary outcome measure was adverse events, graded according to the Common Terminology Criteria for Adverse Events version 4.3. CRS was graded according to the approach described by Lee et al. [17]. GvHD was defined according to criteria established by the Mount Sinai Acute GvHD International Consortium. The secondary outcomes were objective response rate, response duration, progression-free survival, and overall survival. The objective response rate was defined as the proportion of patients achieving a complete response or a complete response with incomplete hematological recovery after the first (*n*)U-CAR-T19 cell infusion before allogeneic stem cell transplantation. Progression-free survival was defined as the time from first (*n*)U-CAR-T19 cell infusion to disease progression, relapse, or death. Overall survival was defined as the time from the first (*n*)U-CAR-T19 cell infusion to death due to any cause. Cytokine levels, CAR-T cell persistence, and CAR copies were measured to evaluate the immune response in vivo.

### Statistics

Statistical analyses were performed using the SPSS17.0 software. For comparisons between two groups, an unpaired *t* test or paired *t* test was used. For comparisons of three or more groups, values were analyzed using one-way ANOVA. (*P < 0.05, **P < 0.01, ***P < 0.001, n.s, no significance).

## Results

### Phenotype and antitumor function of U-CAR-T19 cells* in vitro*

Gene-modified allogeneic CAR-T19 (U-CAR-T19) cells were produced using CRISPR/Cas9 by editing B2M and TRAC on a second-generation backbone containing 4-1BB and CD3ζ intracellular signaling domains (Fig. 1z). After enrichment, the U-CAR-T19 cells were CD3/B2M-negative T cells. Representative enriched U-CAR-T19 cells are shown in Fig. 1b. Lymphocyte subpopulations and inhibitory molecules on CAR-T cells were analyzed using flow cytometry. The results showed that the CD4/CD8 ratio was near 1:1, and CAR^+^ T cells were dominant among CD45RA^+^/CCR7^+^ naïve T cells. PD-1, Tim-3, and LAG3 were expressed at low levels, whereas CD69 was highly expressed in U-CAR-T19 cells (Fig. 1c). Cytotoxicity was comparable between CAR-T19 and U-CAR-T19 cells (Fig. 1D,* P* > 0.05). The anti-leukemic effects of CAR-T19 and U-CAR-T19 cells were compared, and little discrepancy was observed in specific lysis at different E/T ratios, even after double or triple antigen stimulation at E/T of 1:1 (Fig. 1e,* P* > 0.05). IFN-γ and TNF-α secretion after co-culture of CAR-T19 or U-CAR-T19 cells and target cells were comparable (Fig. 1f). In addition, PBMCs from patients with B-ALL and U-CAR-T19 cells were co-cultured at different ratios, and the number of CD19^+^ B cells declined dramatically after 48 h (Supplementary Fig. 1, *P* < 0.05). These results indicated that the specific lysis of U-CAR-T19 cells to leukemia was minimally influenced by gene editing.

### Antitumor ability and alloreactivity of gene-edited CAR-T19 cells* in vivo*

**Fig. 2:**
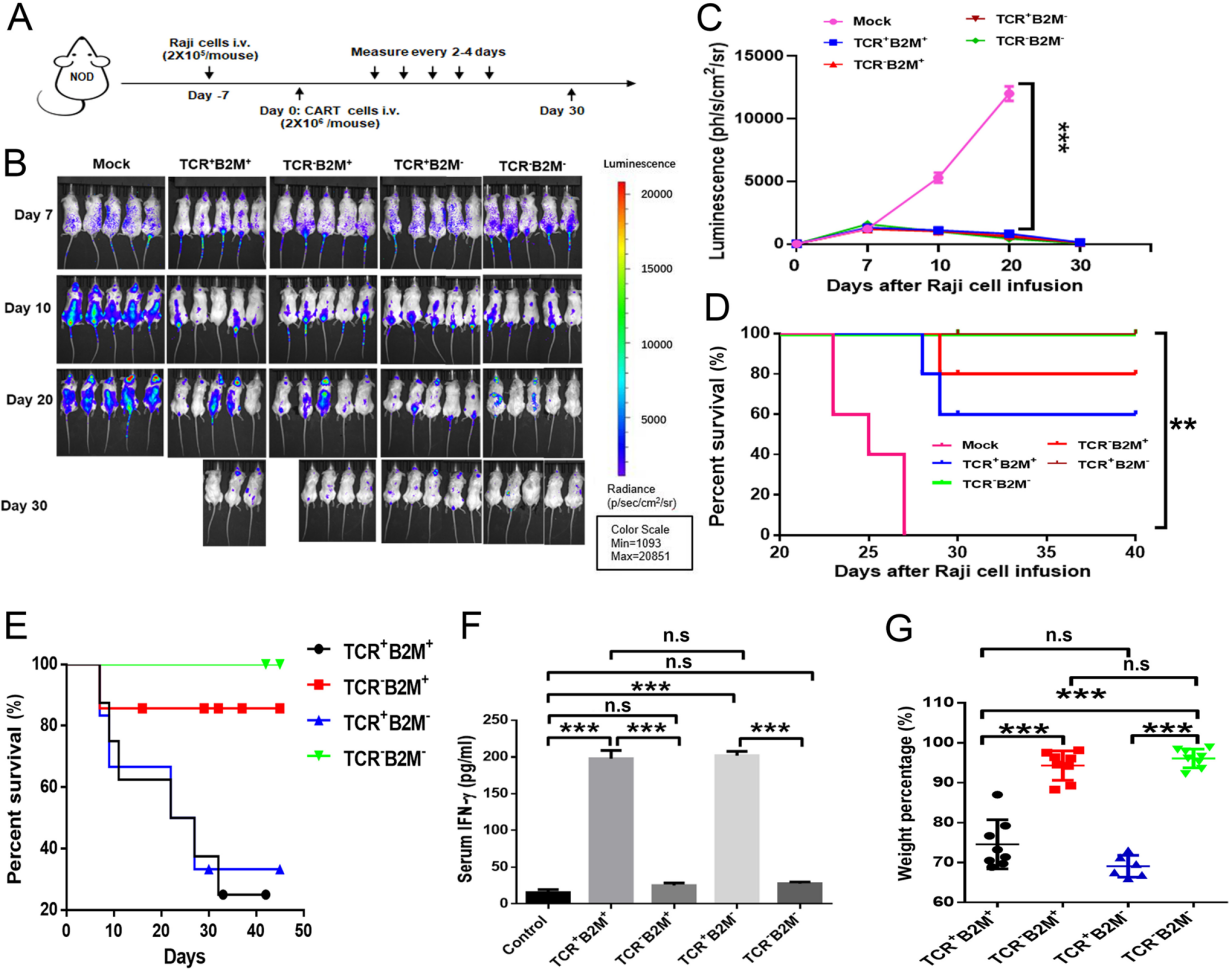
Antitumor ability of U-CAR-T19 cells in an animal model. **a** Schematic of the Raji xenograft tumor model; 8-week-old B-NDG (NOD.CB17-PrkdcscidIl2rgtm1/Bcgen) male mice were inoculated intravenously with ffLuc-transduced Raji cells (2 × 10^5^ cells/mouse), and 2 × 10^6^ *TRAC* or *B2M*-gene-edited CAR-T cells were administered intravenously after 7 days (*n* = 5 mice per group). The IVIS Lumina II In Vivo Imaging System was used to monitor tumor growth every 2–4 days. **b** Fluorescence of each group mice treated with different gene-edited CAR-T19 cells. **c** Averages of bioluminescence in each group mouse treated with different CAR-T19 cells. **d** The survival of each group of mice was assessed, CAR-T cells treated groups had a prolonged survival. **e**–**g** The NSG mice were treated with different gene-edited CAR-T19 cells for the GVHD assay. Survival (**e**), serum IFN-γ (**f**), and body weight change (**g**) of treated mice were assessed. Statistical significance, ANOVA with Tukey’s multiple comparison test or two-tailed, paired Student’s *t* test were used, n.s, no significance, *P < 0.05, **P < 0.01, ***P < 0.001

Based on our in vitro observations, the antitumor activities of the gene-edited CAR-T cells were evaluated using a mouse xenograft lymphoma model. First, 2 × 10^5^ Luc-Raji cells were injected into each mouse, followed by intravenous infusion of each group (*n* = 5) with 2 × 10^6^ gene-edited CAR-T cells via the tail vein (Fig. 2a). The results showed that U-CAR-T19 cells induced the remission of leukemia cells in animal model (Fig. 2b). The averages of bioluminescence in each group mouse treated with different CAR-T19 cells are shown in Fig. 2C (*P* < 0.001), and U-CAR-T19 cells significantly prolonged the survival of tumor-bearing mice (Fig. 2d, P < 0.01). As shown in Fig. 1, the TCR/CD3 complex was significantly ablated by *TRAC* disruption, which is essential for preventing GvHD. To evaluate the potential GvHD risk of U-CAR-T19 cells, CAR-T19 cells, with or without TCR and B2M, were infused intravenously (5 × 10^6^ cells/mouse) after sublethal X-ray irradiation of NOD mice (9 Gy/mouse). The survival of the infused mice was monitored every 2 days for the following 2 months. As shown in Fig. 2e, the mice infused with TCR^+^ cells began to die on day 7, and > 70% of the mice died within 1 month. Accordingly, the secretion of IFN-γ in TCR knockout (KO) cell-treated mice was reduced compared to that in TCR^+^ cell-treated group (Fig. 2f,* P* < 0.001). In addition, the body weight of the *TCR*^*−*^*/B2M*^*−*^ CAR-T19 cell-infused group was little changed (Fig. 2g,* P* < 0.05). All mice survived, and lower levels of IFN-γ and less body weight change in the *TCR/B2M*-negative CAR-T19 cell-treated group, suggested that the GvHD reactivity of genetically-disrupted T cells was abrogated. Taken together, the disruption of TCR and B2M eliminated alloreactivity but retained the antitumor ability of CAR-T cells, demonstrating the safety and effectiveness of U-CAR-T19 cells in clinical applications.

### Patients’ characteristics

Between November 2018 and December 2020, ten patients were screened for inclusion, and nine patients were enrolled and treated with genetically edited allogeneic CAR-T19 cells. Patients' characteristics are shown in Supplementary Table 1. The treatment before enrollment and the major therapeutic process in this clinical trial were established for each patient. In addition, we collected related HLA types of some patients and donors in our study, patients 6 and 7 received U-CAR-T19 cells and nU-CAR-T19 cells prepared from the same donor, the patients 8 and 9 received nU-CAR-T19 cells prepared from donors 6 and 7, respectively (Supplementary Table 2).

### Adverse events

The most common adverse events associated with U-CAR-T19/nU-CAR-T19 cells or lympho-depletion were cytopenia, CRS, and infection. Grade 3 or higher cytopenia (neutropenia or thrombocytopenia) that had not resolved by day 14 was observed in five patients. Neurotoxicity and GvHD were not observed in any patient after the infusion of (*n*)U-CAR-T19 cells. No treatment-related death occurred during the study period. The side effects of pre-chemotherapy and (*n*)U-CAR-T19 cells are shown in Table 1.

**Table 1:** Side effects associated with (*n*)U-CAR-T19 cell infusion

Patient no	Fever	Transaminase	Hypotension	Infection	Abnormalities of coagulation	Thrombocytopenia	Granulocytopenia	Anemia	CRS
1	1	2	0	0	1	4	4	2	2
2	1	0	0	0	0	2	3	2	1
3	1	0	0	0	0	1	4	1	1
4	1	0	0	0	1	1	4	2	1
5	1	0	0	0	1	4	4	3	1
6	1	0	0	0	1	4	4	2	1
7	1	2	0	1	1	4	4	2	2
8	1	0	0	0	1	4	4	2	1
9	1	2	0	1	1	4	4	2	2

### Inefficiency and lack of expansion of U-CAR-T19 cells in patients with B cell malignancies

**Fig. 3:**
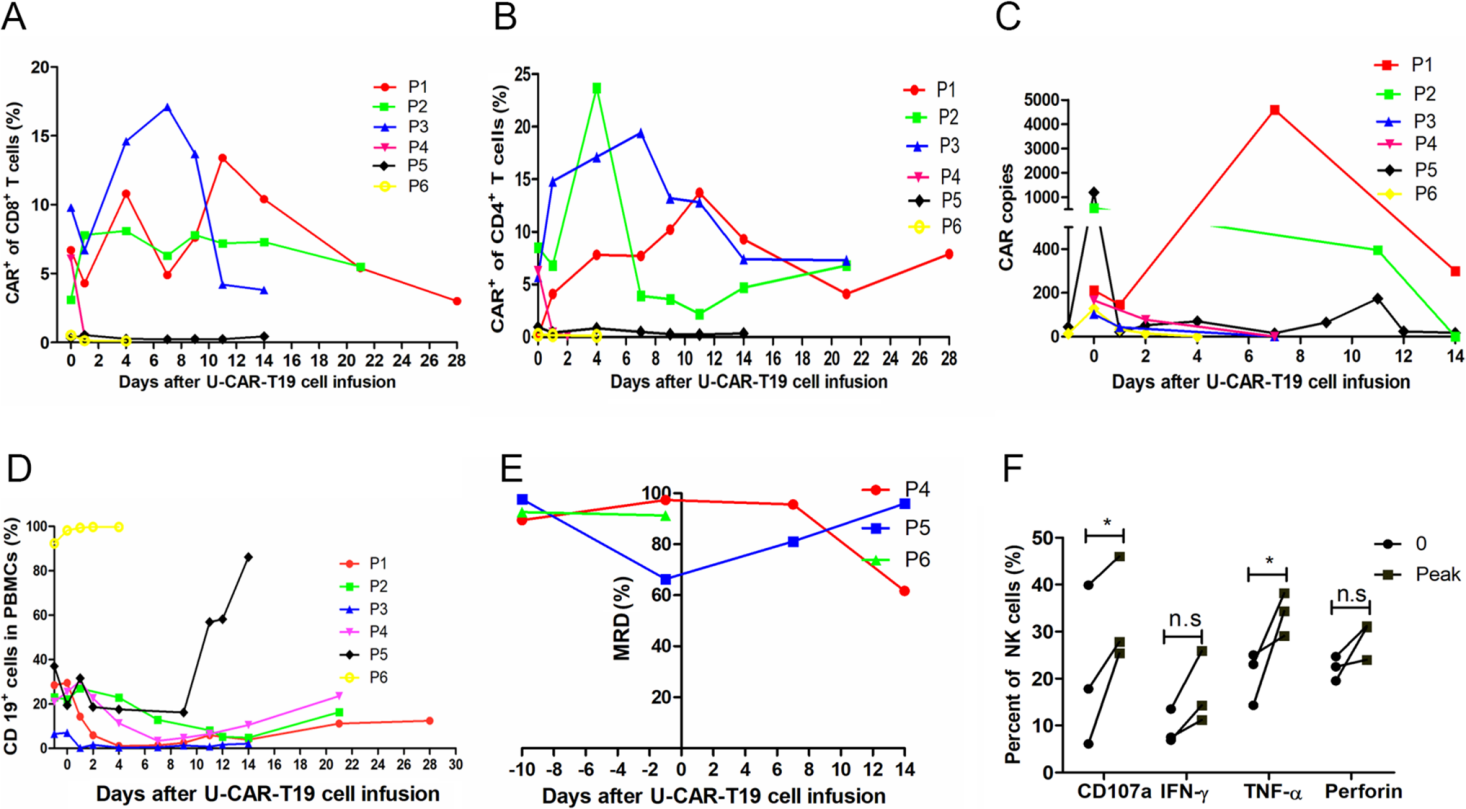
No expansion or antitumor efficacy of U-CAR-T19 cells in patients with R/R B cell malignancies. **a**-**b** Persistence of CAR^+^ CD8^+^ or CD4^+^ cells in vivo after U-CAR-T19 cell infusion. **c** CAR copies from each patient after U-CAR-T19 cell infusion. A qPCR assay was used to detect the CAR mRNA level in vivo. **d** Change in CD19^+^ cells after U-CAR-T19 cell infusion in vivo. **e** MRD of each patient with B-ALL after U-CAR-T19 cell infusion. **f** NK cell activation in vivo after U-CAR-T19 cell infusion. The CD107a, IFN-γ, TNF-α, and perforin expressions in NK cells were analyzed before and after U-CAR-T19 cell infusion in 14 days. The peak values were compared to the base lines. Statistical significance, two-tailed paired Student’s *t* test were used, *P < 0.05

The immune response and persistence of U-CAR-T19 cells were evaluated in three patients with diffuse large B cell lymphoma and three patients with B-ALL. CAR-T cell persistence, CAR copy number, and abnormal B cells were analyzed after the infusion of U-CAR-T19 cells. However, no expansion or antitumor response was observed in the enrolled patients after infusion of U-CAR-T19 cells. The percentages of CAR^+^/CD8^+^ and CAR^+^/CD4^+^ cells in vivo after U-CAR-T19 cell infusion are shown in Fig. 3a and b. The CAR copies of each patient in vivo without notable expansion are shown in Fig. 3c. The percentages of CD19^+^ B cells increased 14 days after U-CAR-T19 cell infusion in vivo (Fig. 3d). Abnormal B cell numbers rapidly increased in the peripheral blood of patients with B-ALL, and hyper-leukocyte leukemia (150 × 10^9^/L) was observed in patient 6 on day 4 after U-CAR-T19 cell infusion. The persistence of CAR and abnormal B cells in the bone marrow of a patient with lymphoma is shown in Supplementary Fig. 2A. PET-CT showed disease progression 1 month after U-CAR-T19 cell infusion in patients 1 and 2 (Supplementary Fig. 2B). The enlarged lymph nodes of the neck progressed rapidly in the patient 3 in 14 days after U-CAR-T19 cell infusion. In addition, three patients with R/R B-ALL received U-CAR-T19 cell infusion, and the MRD in the bone marrow did not decline after 14 days (Fig. 3e). However, U-CAR-T19 cells induced the specific lysis of primary leukemia cells from leukemia patients in vitro. These results indicated that the anti-leukemic ability of U-CAR-T19 cells was limited by the lack of in vivo expansion, suggesting the potential immune rejection of allogeneic U-CAR-T19 cells by the host. However, anti-CAR immune responses are complex, and anti-CAR antibodies can potentially induce the death of CAR-T cells via several mechanisms, including cytotoxicity via activated immune cells [18]. To determine the reason for the absence of U-CAR-T19 cells in patients, dynamic changes in immune cells were detected before and after U-CAR-T19 cell infusion. NK cells were significantly activated and expanded following the elimination of U-CAR-T19 cells. The percentage of CD107a^+^ or TNF-α^+^ NK cells increased significantly 14 days after U-CAR-T19 cell infusion (Fig. 3F,* P* < 0.05). B2M KO completely disrupt all structures of HLA-I, including C and E antigens, which are considered inhibitory ligands to NK cells, thus, *B2 M*^*−*^*/TRAC*^*−*^ T cells are sensitive to allogeneic NK cell-mediated killing [19, 20]. HLA-C and HLA-E expressions are involved in mediating the resistance of T cells to NK cells [21, 22]. Thus, novel U-CAR-T19 (nU-CAR-T19) cells were generated by editing *HLA-A, HLA-B*, and *TRAC* using CRISPR/Cas9 technology while preserving other HLA-I molecules.

### Feasibility of nU-CAR-T cells

**Fig. 4:**
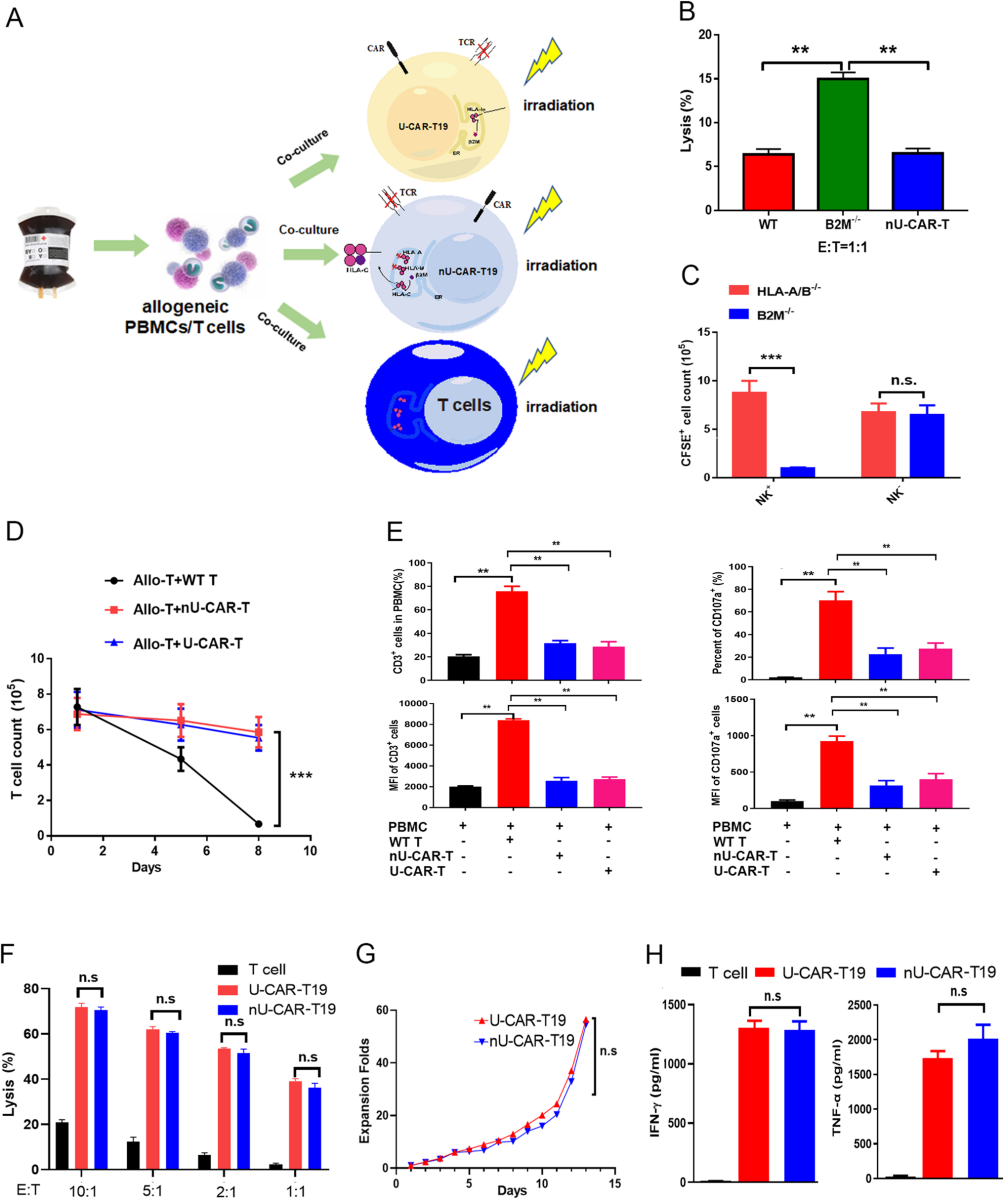
Characteristics of *HLA-A*^*−*^*/HLA-B*^*−*^* TRAC*^*−*^ CAR-T19 (nU-CAR-T19) cells. **a** In order to investigate the immunogenicity of gene modified CAR-T cells to host immune system, the allogeneic PBMCs as well as T cells were isolated from the health donor to co-culture with irradiated donor T, nU-CAR-T19 and U-CAR-T19 cells to perform the cytotoxicity assay. **b** WT T, U-CAR-T19, and nU-CAR-T19 cells were co-cultured with PBMCs from the same donor, among which WT T, U-CAR-T19, and nU-CAR-T19 cells were stained with CFSE fluorescence before co-cultured with allogeneic PBMCs at the effective target ratio of 1:1. The residual proportion of CFSE-positive cells was detected on the days 3 and 6. **c** The nU-CAR-T19 and U-CAR-T19 cells were pre-stained with CFSE, and then co-cultured with allogeneic PBMCs with or without NK cells (NK cells were removed with magnetic beads to eliminate their function) in the E/T ratio of 1: 1 for 24 h. The residual (*n*)U-CAR-T19 cells of CFSE^+^ were detected and analyzed using flow cytometry. **d** WT T, nU-CAR-T19 and U-CAR-T19 cells were pre-stained with CFSE and irradiated with 30 Gy X-Ray to avoid self-proliferation and activation, and then co-cultured with CD3/CD28 antibody-activated allogeneic T cells for 24 and 72 h. The CFSE^+^ cells were detected using flow cytometry. **e** No activation of allogeneic T cells stimulated with nU-CAR-T19 cells was observed. WT T, nU-CAR-T19 and U-CAR-T19 cells were pre-stained with CFSE and irradiated with 30 Gy X-Ray, and then co-cultured with allogeneic PBMCs for 7 days. The expansion (CD3^+^) and activation (CD107a^+^) of allogeneic T cells were detected using flow cytometry. The percentages and MFI of CD3^+^ and CD107a^+^ T cells in different groups were compared to that of WT T cell group.** f** U-CAR-T19 and nU-CAR-T19 cells were stimulated with Raji cells at the given E/T ratio. The killing of effector cells to target cells was detected by measuring the release of lactate dehydrogenase (LDH). **g** U-CAR-T19 and nU-CAR-T19 cell proliferation in response to stimulation with CD19-expressing Raji cells. **h** U-CAR-T19 and nU-CAR-T19 cells were stimulated with CD19-expressing Raji cells at E/T ratio of 1:1 for 24 h. IFN-γ and TNF-α expressions were determined by ELISA. These experiments were repeated three times with similar results. Statistical significance, ANOVA with Tukey’s multiple comparison test or two-tailed, paired Student’s *t* test were used, n.s, no significance, *P < 0.05, **P < 0.01, ***P < 0.001

The highly polymorphic *HLA-A* and *HLA-B* genes were knocked out in nU-CAR-T19 cells, and only HLA-A and HLA-B were deleted, as confirmed using flow cytometry. The results showed that HLA-A and HLA-B expressions in the edited cells decreased significantly (Supplementary Fig. 3A). WT T, U-CAR-T19, and nU-CAR-T19 cells were co-cultured with PBMCs from the same donor, among which T, U-CAR-T19, and nU-CAR-T19 cells were stained with CFSE fluorescence in advance and co-cultured with allogeneic PBMCs at an effective target ratio of 1:1 to perform the cytotoxicity assay (Fig. 4a). Residual proportions of CFSE-positive cells were detected on days 3 and 6 (Supplementary Fig. 3B). The results showed that U-CAR-T19 cells were eliminated by allogeneic PBMCs. However, WT T and nU-CAR-T19 cells were slightly lysed (Fig. 4b,* P* < 0.01), indicating that nU-CAR-T19 cells had a stronger advantage for further studies.

### nU-CAR-T cells resisted allogeneic NK cells

To further confirm the clearance of allogeneic CAR-T19 cells, the nU-CAR-T19 and U-CAR-T19 cells were pre-stained with CFSE, and co-cultured with allogeneic PBMCs with and without NK cells at an effective target ratio of 1: 1 for 24 h. The residual nU-CAR-T19 cells of CFSE^+^ were detected using flow cytometry (Supplementary Fig. 3C). The results showed that NK cells quickly eliminated U-CAR-T19 cells, whereas nU-CAR-T19 cells were retained (Fig. 4c,* P* < 0.001). In contrast, nU-CAR-T19 and U-CAR-T19 cells co-cultured with allogeneic PBMCs without NK cells did not show any difference (Fig. 4c,* P* > 0.05). These results indicated that NK cells play an important role in mediating the immune rejection of allogeneic cells with decreased expression of HLA-I molecules, whereas nU-CAR-T19 cells were significantly resistant to the rejection of allogeneic NK cells.

### nU-CAR-T cells resisted the rejection of activated T cells

WT T, nU-CAR-T19, and U-CAR-T19 cells were pre-stained with CFSE, irradiated with 30 Gy X-rays, and co-cultured with CD3/CD28 antibody-activated allogeneic T cells for 24 and 72 h. Residual CFSE^+^ cells were detected using flow cytometry (Supplementary Fig. 3D). The results showed that nU-CAR-T19 and U-CAR-T19 cells were resistant to rejection of activated allogeneic T cells (Fig. 4d,* P* < 0.001). WT T, nU-CAR-T19, and U-CAR-T19 cells were pre-stained with CFSE, irradiated with 30 Gy X-rays, and then co-cultured with allogeneic PBMCs for 7 days. The expansion and activation of CD3^+^ allogeneic T cells (CD107a^+^) were detected using flow cytometry (Supplementary Fig. 3e). The results showed that WT T cells activated CD3^+^ T cells in allogeneic PBMCs and promoted their expansion, whereas nU-CAR-T and U-CAR-T19 cells significantly reduced the activation of allogeneic T cells compared to those of WT T cell groups (Fig. 4e,* P* < 0.01). These results indicated that both nU-CAR-T19 and U-CAR-T19 cells had a good immune tolerance to allogeneic T cells.

### nU-CAR-T19 cells exhibited equivalent antitumor ability compared to U-CAR-T19 cells

To evaluate the antitumor abilities of CAR-T cells in vitro, WT T, U-CAR-T19, and nU-CAR-T19 cells were co-cultured with Raji cells at different ratios for 24 h, and specific lysis was detected by measuring the release of LDH. The results showed that nU-CAR-T19 cells displayed equivalently antitumor activity to that of U-CAR-T19 cells (Fig. 4f,* P* > *0.05*). Accordingly, a similar expansion was observed between U-CAR-T19 and nU-CAR-T19 cells stimulated with target cells at a ratio of 1:1 (Fig. 4g,* P* > *0.05*). In addition, IFN-γ and TNF-α levels were equivalent between U-CAR-T19 and nU-CAR-T19 cells stimulated with Raji cells at a ratio of 1:1 for 24 h (Fig. 4h,* P* > *0.05*). These results indicated that the function of nU-CAR-T19 cells was not affected by *HLA-A/B* and *TRAC* disruption.

### nU-CAR-T19 cells produce potential anti-leukemia ability in patients with R/R B-ALL

**Fig. 5:**
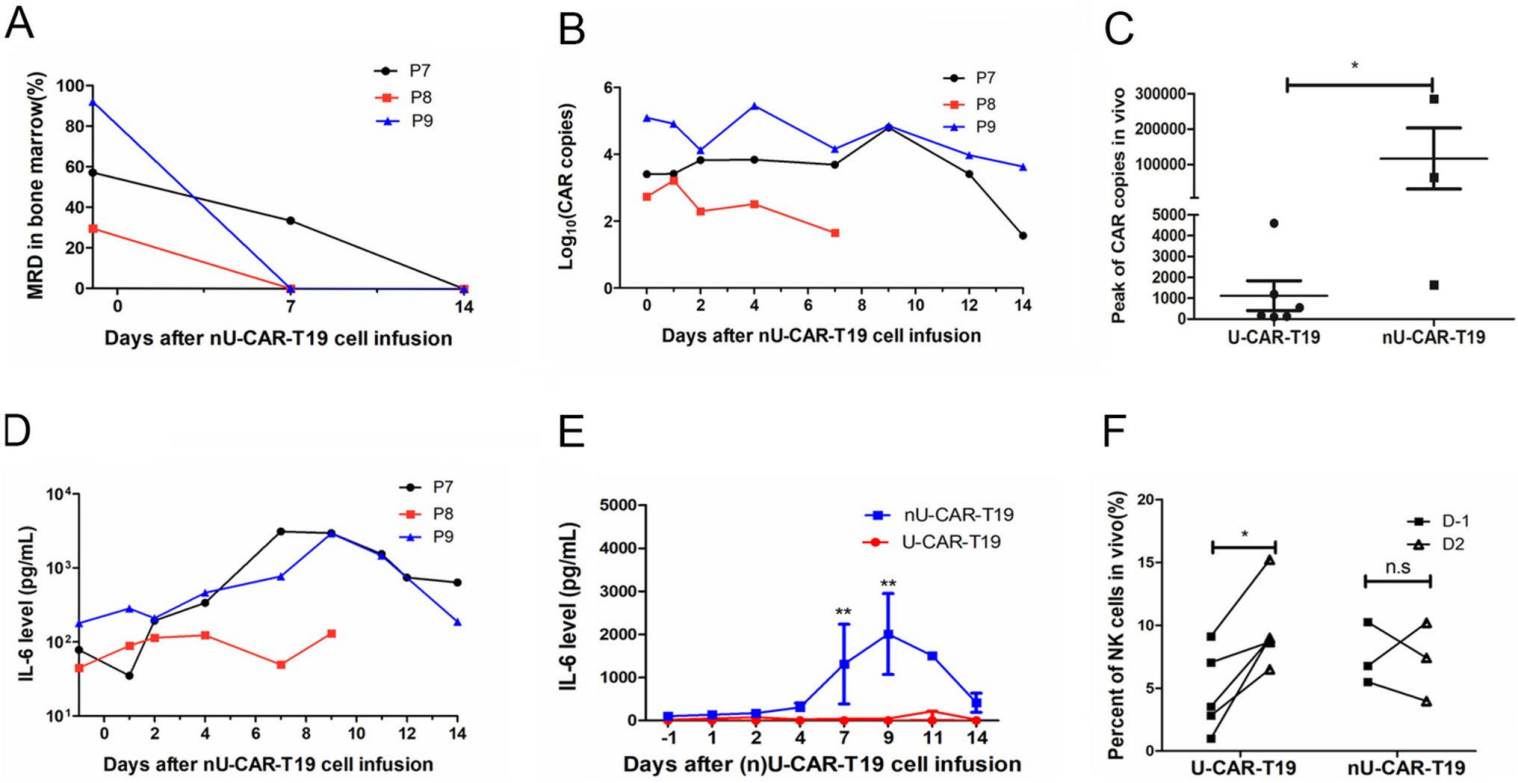
Anti-leukemia ability of nU-CAR-T19 cells in patients with R/R B-ALL. **a** MRD of bone marrow in patients with R/R B-ALL after infusion of nU-CAR-T19 cells. **b** The persistence and expansion of nU-CAR-T19 cells in vivo*.* **c** Compared to patients treated with U-CAR-T19 cells, a higher peak in CAR copy number was observed in patients infused with nU-CAR-T19 cells. **d** Dynamic changes in IL-6 levels in vivo after the infusion of nU-CAR-T19 cells. **e** Higher IL-6 levels were observed in patients treated with nU-CAR-T19 cells than in patients treated with U-CAR-T19 cells. **f** No NK cell activation after infusion of nU-CAR-T19 cells. The percentages of CD16^+^/CD56^+^ cells in patients treated with (*n*)U-CAR-T19 cells in vivo were analyzed using flow cytometry. After U-CAR-T19 cell infusion, the percentage of NK cells increased (*n* = 5). Statistical significance, two-tailed, non-paired or paired Student’s *t* tests were used, n.s, no significance, *P < 0.05, **P < 0.01

Basic resistance to other immune cells of nU-CAR-T19 cells and the antitumor activity of nU-CAR-T19 cells in clinical were further explored. Three patients with R/R B-ALL received nU-CAR-T19 cell infusion derived from three donors after pre-chemotherapy composed of fludarabine, cyclophosphamide and etoposide, and total body irradiation of 2 Gy × 3. All patients achieved MRD negativity 14 days after nU-CAR-T19 cell infusion (Fig. 5a). The persistence and expansion of nU-CAR-T19 cells were detected using qPCR (Fig. 5b). Compared to the U-CAR-T19 cells in patients with R/R B cell malignancies, nU-CAR-T19 cells had a higher peak of expansion in vivo (Fig. 5c,* P* < 0.05). Patients 7 and 9 experienced grade 2 CRS, accompanied by CAR-T cell expansion. Dynamic IL-6 levels in each patient are shown in Fig. 5D. Compared to those in patients treated by U-CAR-T19 cells, IL-6 levels were higher in patients treated with nU-CAR-T19 cells (Fig. 5e,* P* < 0.01). The percentage of CD16^+^ or CD56^+^ NK cells in vivo was analyzed using flow cytometry, which indicated that NK cells were not significantly activated or expanded after infusion with nU-CAR-T19 cells (Fig. 5f). Myelosuppression, granulocytopenia, thrombocytopenia, and anemia were observed in all the patients (Table 1). Patients 7 and 9 suffered grade 2 of CRS, which were managed with an IL-6 receptor blocker. GvHD and neurotoxicity were not observed in the patients treated with nU-CAR-T19 cells. These results showed that nU-CAR-T19 cells had effective and safe anti-leukemic activity in patients in vivo.

### Patient 7 achieved MRD negativity twice after two infusions of the same batch of nU-CAR-T19 cells

**Fig. 6:**
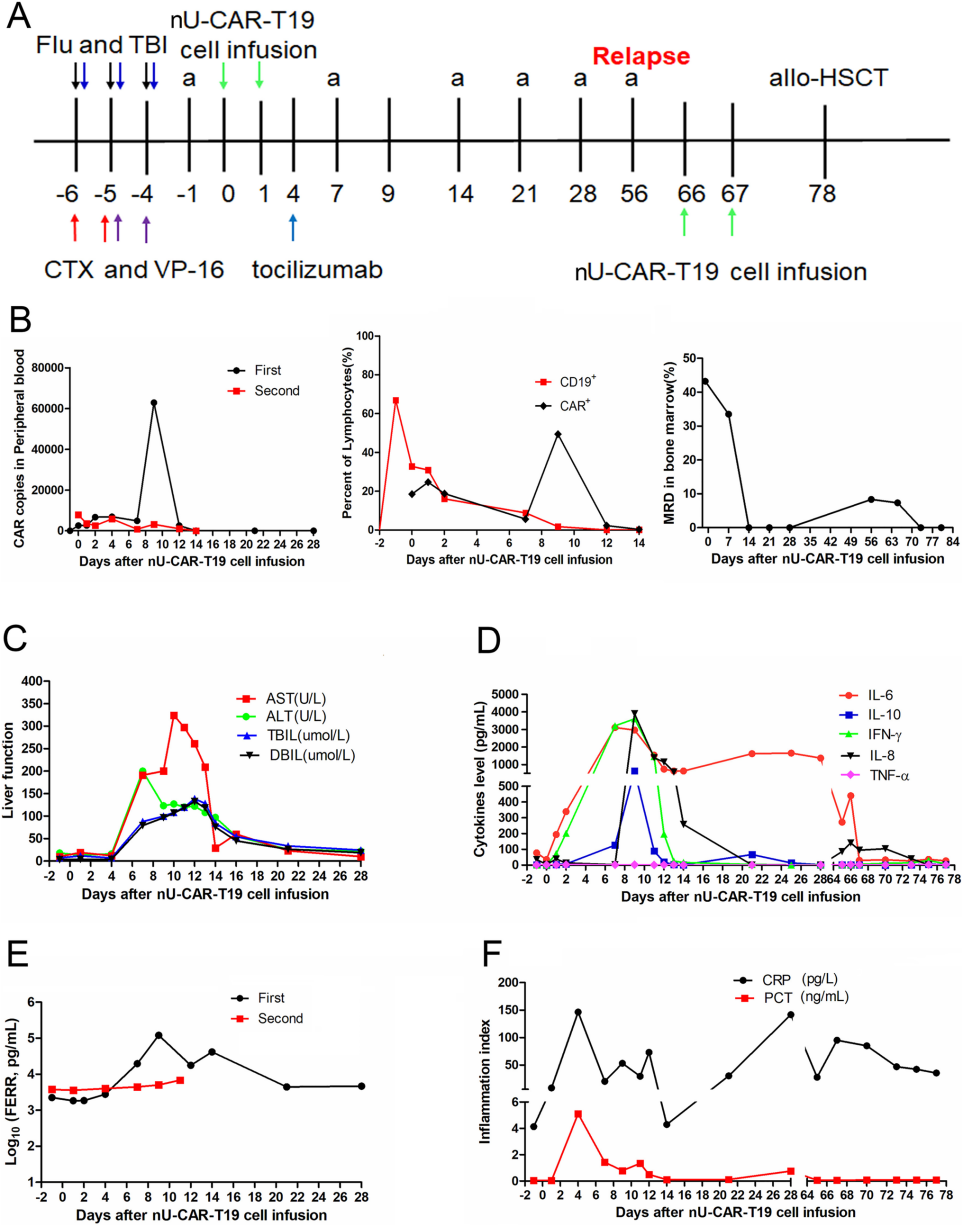
A patient with R/R B-ALL obtained MRD negativity twice after two round infusions of nU-CAR-T19 cells. **a** Major treatment progress before and after the infusion of nU-CAR-T19 cells. The patient received fludarabine (Flu, 25 mg/m^2^, D-6 to D-4), cyclophosphamide (CTX, 500 mg/m^2^, D-6 to D-5), and etoposide (VP-16, 100 mg/day, D-5 to D-4) and TBI (2 Gy/time, D-6 to D-4). Tumor burden was evaluated through bone marrow biopsy (**a**) at the D-1, every week in 1 and 2 months after nU-CAR-T19 cell infusion. MRD was negative at 14 days and again positive at 56 days after the first infusion of nU-CAR-T19 cells. Then, patient 7 achieved negative MRD after receiving the second infusion of the same bath nU-CAR-T19 cells (D66-67) for 7 days and later accepted the allo-HSCT. **b** Dynamic changes in CAR persistence and CD19^+^ B cells in vivo after infusion of nU-CAR-T19 cells. **b** Changes in live function in vivo after the first infusion of nU-CAR-T19 cells. **d** Dynamic changes in cytokine levels in vivo after the infusion of nU-CAR-T19 cells. **e** Dynamic changes in FERR level in vivo after the infusion of nU-CAR-T19 cells. **f** Dynamic changes in the inflammation index in vivo after the infusion of nU-CAR-T19 cells

Patient 7 was diagnosed with B-ALL that was refractory to multiple lines of chemotherapy. The therapeutic processes used in the selected clinical trials are shown in Fig. 6a. The patient received pre-chemotherapy and total body irradiation of 2 Gy × 3 before nU-CAR-T19 cell infusion. The CAR copy number and percentage of CAR-T cells peaked on 9 days after nU-CAR-T19 cell infusion, and CD19^+^ B cells in PBMCs and bone marrow declined 14 days after nU-CAR-T19 cell infusion (Fig. 6b). After the first infusion with nU-CAR-T19 cells, patient 7 had continuous fever for 3 days and suffered from grade 2 CRS and hepatotoxicity, which recovered after symptomatic treatment for 21 days without any neurotoxicity or GvHD (Fig. 6c). IL-6, IL-8, IL-10, IFN-γ, TNF-α, and ferroprotein (FERR) levels peaked at 9 days after the first infusion with nU-CAR-T19 cells (Fig. 6d and e). However, secondary perianal infection occurred due to chronic grade 4 myelosuppression, and CRP and PCT levels are shown in Fig. 5f. The patient achieved MRD negativity at 14 days but relapsed with CD19 antigen positivity at 56 days after nU-CAR-T19 cell infusion. The MRD again turned negative 7 days after receiving the same batch of nU-CAR-T19 cells (CAR^+^ 6.0 M/kg) after a pre-chemotherapy regimen comprising of fludarabine, cyclophosphamide, and etoposide for 2 days. CAR copies in the peripheral blood after the second infusion were detected by qPCR and are shown in Fig. 6b. Side effects of the second infusion included slight myelotoxicity and fever. Cytokines, FERR, PCT, and CRP levels are summarized in Fig. 6d–f. Then, she underwent allogeneic hematopoietic stem cell transplantation. These results indicated that nU-CAR-T19 cells exhibited manageable safety and prominent “off the shelf” anti-leukemic activity.

## Discussion

The present study aimed to evaluate the feasibility and efficiency of genetically edited allogeneic CAR-T19 cells in patients with R/R B cell malignancies who had previously undergone multiple lines of therapy and were unsuitable for autologous CAR-T cell therapy. The high cost, long-term or failure of preparation, disease progression, high individuality, and lack of chances for apheresis limit the large-scale use of autologous CAR-T cells [23, 24]. U-CAR-T cells are currently being explored in the field of cancer immunotherapy [8]. Multiple gene-editing methods and other types of immune cells for CAR-T cell products have been attempted [25]. There are two major issues with “off the shelf” allogeneic CAR-T cells: (1) GvHD is a potential side effect of their use in vivo and (2) rejection by the host immune system limits their persistence, expansion, and antitumor function. GvHD is confirmed in HLA mismatches and primarily caused by αβT cells [26, 27]. The prevention of GvHD via TCR KO in U-CAR-T cells using different strategies has been reported. *TRAC* depletion is an important strategy for disrupting the TCR and was first reported in CAR-T19 cells in 2012 [28]. The targets of U-CAR-T cells mainly included CD19, CD7, and CD123 [29–31]. Transcription activator-like effector nuclease (TALEN) or CRISPR/Cas9-edited *TRAC* for CAR-T19 cells exhibited antitumor immune responses in clinicals [32, 33]. Indeed, two infants with R/R B-ALL achieved CR using TALEN-mediated *TRAC*^*−*^ and *CD52*^*−*^ U-CAR-T19 cells [13]. Recently, it was reported that six patients with R/R B-ALL received TT52CAR19T cell infusion, four patients achieved remission, two patients had grade II CRS, and one patient developed skin GVHD [31]. CRISPR/Cas9 disrupted the *TRAC* region and *CD52* gene-edited dual CD19/CD22 CAR-T cells also showed antitumor efficacy in patients with R/R B-ALL; and no CRISPR gene-editing-associated genotoxicity, immunogenicity, or GvHD was observed in this study [34]. Overall, these results support further exploration of novel gene-edited allogeneic CAR-T cells using CRISPR/Cas9 technology. HLA mismatch is a key factor in mediating T cell rejection, and *B2M* editing by CRISPR/Cas9 could reduce HLA-mediated immune rejection [35]. Thus, U-CAR-T19 cells were designed through CRISPR/Cas9-meditated disruption of *TRAC* and *B2M*. The specific lysis, expansion, and antitumor abilities in the animal model of CAR-T19 and U-CAR-T19 cells were equivalent. Although Hayley Virgil reported positive results for phase 1 trial of allogeneic gene-edited CAR-T CTX110 (both *TRAC* and *B2M* were disrupted by CRISPR/Cas9) in R/R CD19^+^ B cell malignancies [25], no expansion or antitumor efficiency of *TRAC*^*−*^*/B2 M*^*−*^-edited U-CAR-T19 cells was observed in patients with R/R B cell malignancies in the current study. NK cell activation was observed after infusion of U-CAR-T19 cells in vivo*.* HLA-E and HLA-G are ligands of NK cell-inhibitory receptors that may prevent NK cell lysis [22, 36, 37]. *B2 M* KO caused the disruption of all class I major histocompatibility complexes (MHC I), which activated NK cell-mediated elimination of allogeneic cells [38]. In addition, it was reported that CRISPR/Cas9-mediated knockout of HLA class I/II and TRAC molecules displayed enhanced resistance against elimination by allogeneic PBMCs; thus, knockout of HLA-II molecule in allogeneic CAR-T cells might improve resistance to CD4^+^ T cells in vivo [39, 40]. In contrast, HLA-C-retained gene-edited immune cells could evade the activity of NK cells [41]. However, the HLA types of enrolled patients and donors were not completely detected in advance in this study. HLA types of some patients and donors were further collected, compared to no expansion and antitumor ability of U-CAR-T19 cells in patient 6, nU-CAR-T19 cells prepared from the same donor produced stronger anti-leukemia ability in patient 7. Patients 7, 8, and 9 received nU-CAR-T19 cells prepared from different donors, HLA-C sites of these patients and donors were partial overlap. Whether matching in HLA-C could increase the persistence of allogenic CAR-T19 cells is need to verified by more clinical data.

In our study, based on the safety of U-CAR-T19 cells in patients with R/R B cell malignancies, at a dose of 5–6 M/kg, nU-CAR-T19 cells produced antitumor activity in several patients with R/R B-ALL. The combined FC, VP-16, and TBI scheme is an option for the pre-treatment of the CAR-T cell therapeutic process. In patients, we observed improved peak expansion over U-CAR-T19 cells, which lacked all HLA class I molecules, indicating that retaining HLA-C/E might be advantageous. This conclusion is further supported by our in vitro analysis, which demonstrated improved NK cell resistance of HLA-C/E^+^ nU-CAR-T19 cells compared to that of U-CAR-T19 cells which lacked all HLA class I molecules. As nU-CAR-T19 cells did not activate NK cells, we observed no increase of activation markers on NK cells in the blood at peak CAR-T cell expansion, which was observed for U-CAR-T19 cells.

Notably, patient 7 achieved MRD negativity twice after the infusion of nU-CAR-T19 cells. The expansion of nU-CAR-T19 cells reached a maximum on day nine with a decrease in the number of abnormal B cells after the first infusion. The patient experienced grade 2 CRS, recovered within 14 days of infusion, and experienced long-term agranulemia and perianal infection. However, relapse is an issue with a CAR-T19 cells for R/R B cell malignancies, and a second infusion of CAR-T19 cells could produce a lower CR rate in R/R B cell malignancies [42]. The patient relapsed with CD19 positivity and again achieved MRD negativity with the infusion of the same batch of nU-CAR-T19 cells. These results supported the idea that the second infusion of genetically edited allogeneic CAR-T cells also produced potential anti-leukemia effects and displayed low immunogenicity. nU-CAR-T19 cells could produce specific anti-leukemic effects and provide a novel strategy for the development of universal CAR-T cells. Future, more clinical data are needed to confirm the better outcome and long-term survival in patients received nU-CAR-T19 cell infusion.

## Conclusions

The expansion and antitumor ability of U-CAR-T19 cells are unsatisfactory in patients with B cell malignancies. NK cell activation and killing mediate the rejection of U-CAR-T19 cells. nU-CAR-T19 cells exhibited safety and anti-leukemia activity in patients with R/R B-ALL. However, further clinical trials are underway.

### Supplementary Information

Below is the link to the electronic supplementary material.

Supplementary file1 (TIF 5768 KB)

Supplementary file2 (TIF 1786 KB)

Supplementary file3 (TIF 10807 KB)

Supplementary file4 (DOCX 15 KB)

Supplementary file5 (DOCX 19 KB)

Supplementary file6 (DOC 33 KB)

## Data Availability

Data are available upon request for academic researchers.

## Abbreviations

B-ALL: Acute B cell lymphoblastic leukemia
CAR: Chimeric antigen receptor
CR: Complete response
CRS: Cytokine release syndrome
E/T: Effector/target
GvHD: Graft-versus-host-disease
KO: Knockout
LDH: Lactate dehydrogenase
MHC I: Major histocompatibility complexes
MRD: Minimal residual disease
R/R: Relapsed or refractory
TRAC: T cell receptor alpha constant
TALEN: Transcription activator-like effector nuclease
WT: Wild-type

## Declarations

### Provenance and peer review

Not commissioned; externally peer reviewed.
