# Supplementary figures and images for: Allogeneic CAR-T cells with of HLA-A/B and TRAC disruption exhibit promising antitumor capacity against B cell malignancies
Source: Cancer Immunol Immunother. 2024 Jan 17;73(1):13. doi: 10.1007/s00262-023-03586-1 (PMC10794471; doi:10.1007/s00262-023-03586-1)

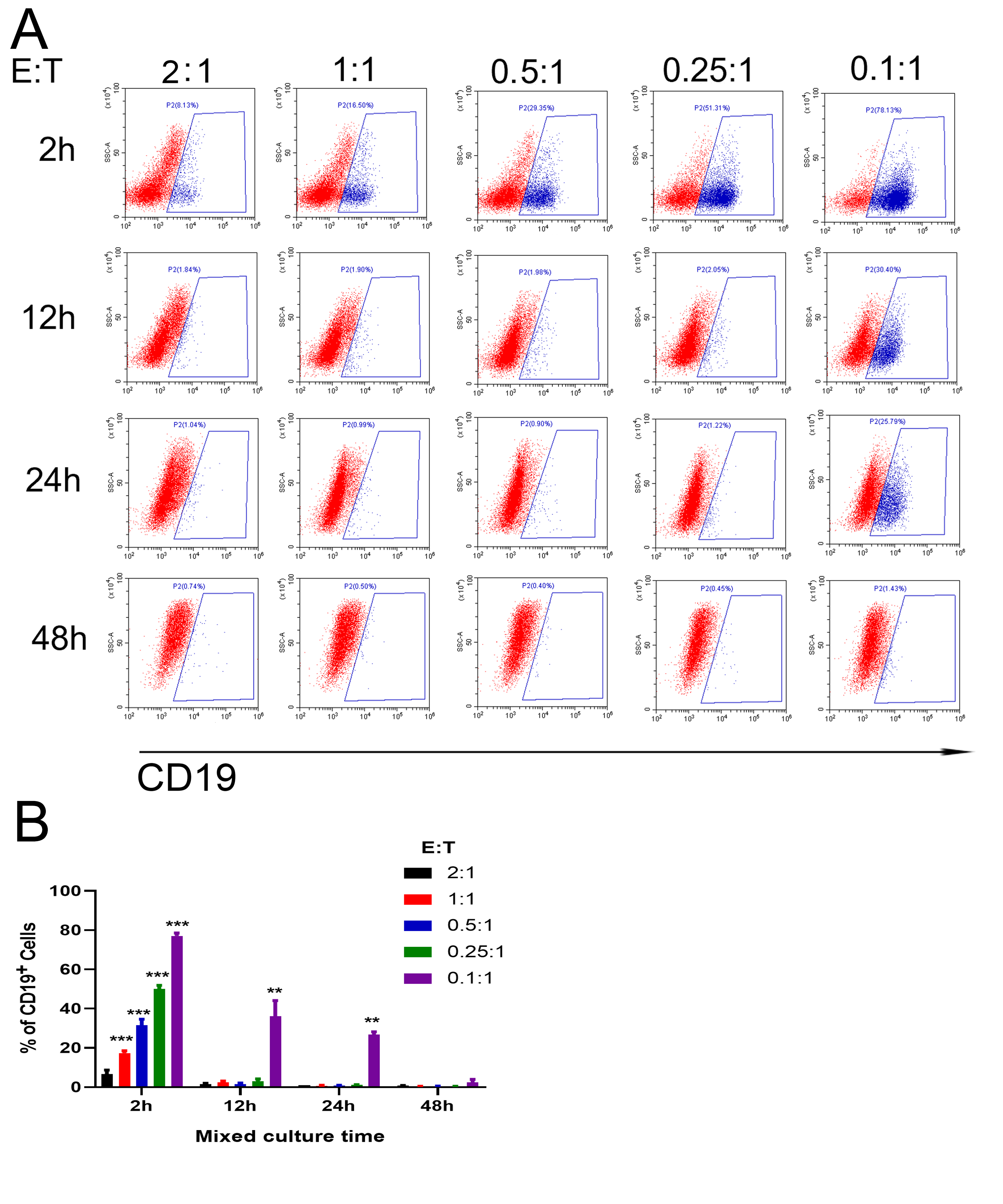

Supplement: Supplementary file 1 — Supplementary file1 (TIF 5768 KB) [file 262_2023_3586_MOESM1_ESM.tif]

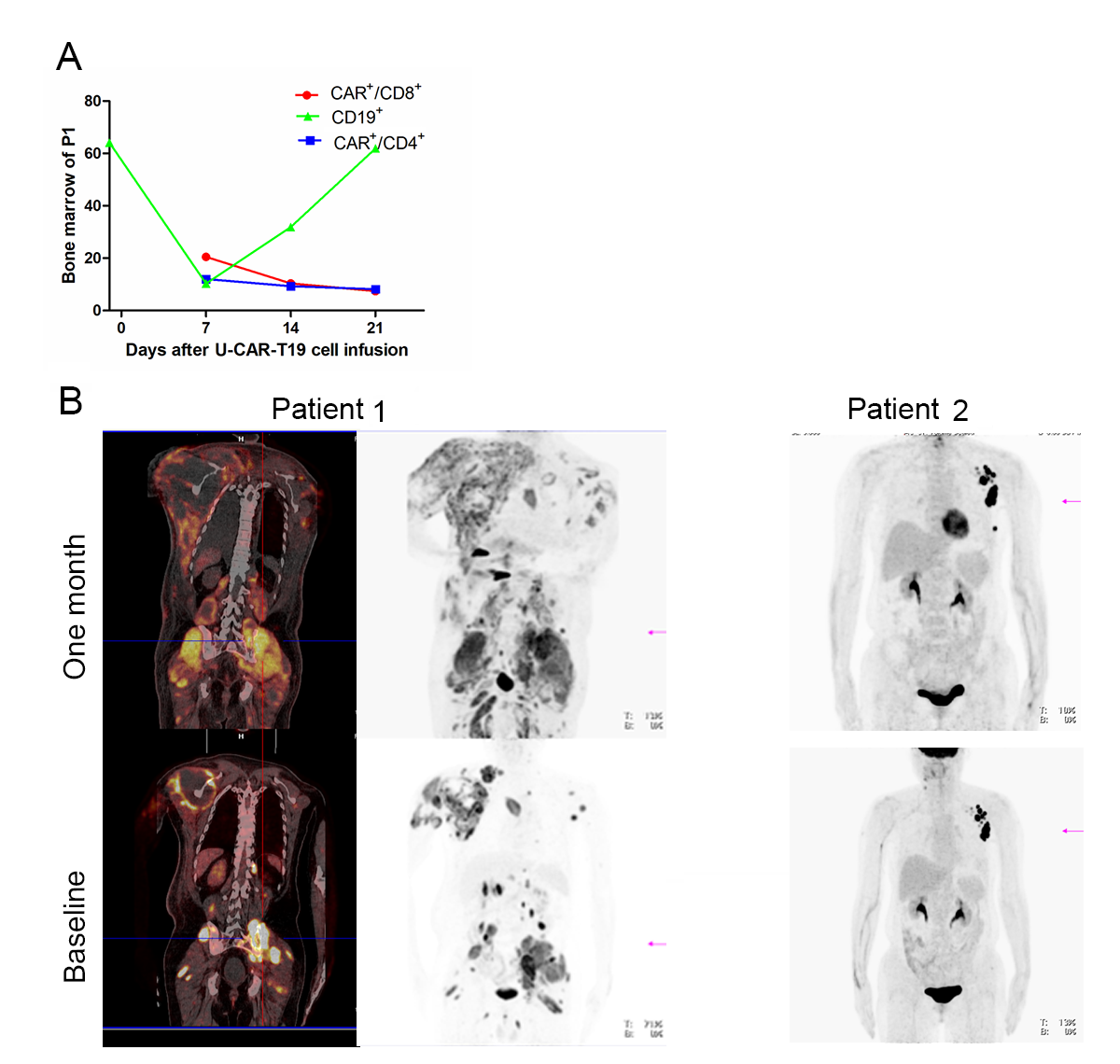

Supplement: Supplementary file 2 — Supplementary file2 (TIF 1786 KB) [file 262_2023_3586_MOESM2_ESM.tif]

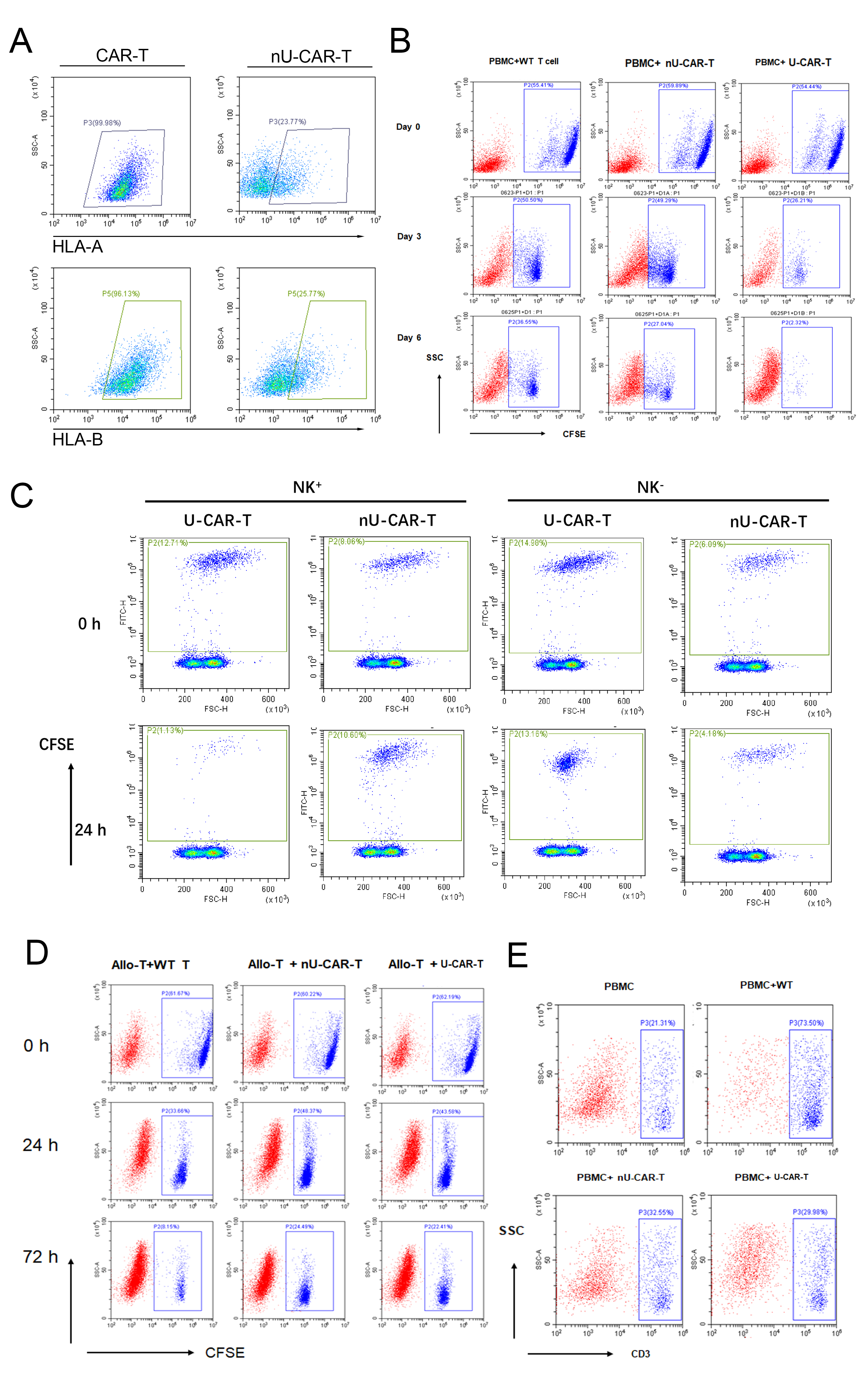

Supplement: Supplementary file 3 — Supplementary file3 (TIF 10807 KB) [file 262_2023_3586_MOESM3_ESM.tif]
