# Supplementary material for: Allogeneic CAR-T cells with of HLA-A/B and TRAC disruption exhibit promising antitumor capacity against B cell malignancies
Source: Cancer Immunol Immunother. 2024 Jan 17;73(1):13. doi: 10.1007/s00262-023-03586-1 (PMC10794471; doi:10.1007/s00262-023-03586-1)
Supplement: Supplementary file 4 — Supplementary file4 (DOCX 15 KB) [file 262_2023_3586_MOESM4_ESM.docx]

**Supplementary Fig 1. Specific lysis of U-CAR-T19 cells stimulated with primary leukemia cells.**

1. Representative images of specific lysis of U-CAR-T19 cells co-cultured with primary

leukemia cells from a patient with R/R B-ALL. Co-culture of nU-CAR-T19 with leukemia cells from B-ALL patients with different effector to target rate (E:T). CD19^+^ residual cells were monitored at the defined timepoints with flow cytometry. Percentage of CD19^+^ cells at 2h was set as control group. (B) U-CAR-T19 cells produced a high specific lysis at different E:T ratios. Two ways ANOVA, ***, *P*<0.001; **, *P*<0.01; *, *P*<0.05.

**Supplementary Fig 2. Disease progression in patients with lymphoma after U-CAR-T19 cell infusion.**

(A) CAR persistence and abnormal CD19^+^ B cells in bone marrow from a lymphoma patient after infusion of U-CAR-T19 cells. (B) PET-CT scan Images of two patients at base line and after treated with U-CAR-T19 cell infusion for one month.

**Supplementary Fig 3. Representative images of nU-CAR-T19 cells compared to U-CAR-T19 cells in different conditions by flow cytometry.**

(A) Expression of HLA-A/B in CAR-T19 cells and nU-CAR-T19 cells. (B) Residual CFSE^+^ WT T cells, nU-CAR-T19 cells, and U-CAR-T19 cells co-cultured with allogeneic PBMCs were analyzed using flow cytometry. (C) Removal of NK cells increased residual U-CAR-T19 cells as analyzed using flow cytometry. (D) Residual CFSE^+^ WT T cells, nU-CAR-T19 cells, and U-CAR-T19 cells co-cultured with allogeneic T cells were analyzed using flow cytometry. (E) Expansion of allogeneic T cells co-cultured with WT T, nU-CAR-T19, and U-CAR-T19 cells were analyzed using flow cytometry.
