# Supplementary material for: Allogeneic CAR-T cells with of HLA-A/B and TRAC disruption exhibit promising antitumor capacity against B cell malignancies
Source: Cancer Immunol Immunother. 2024 Jan 17;73(1):13. doi: 10.1007/s00262-023-03586-1 (PMC10794471; doi:10.1007/s00262-023-03586-1)
Supplement: Supplementary file 5 — Supplementary file5 (DOCX 19 KB) [file 262_2023_3586_MOESM5_ESM.docx]

**Supplementary Table 1 Characteristics of treated patients**

| Patient  No. | Diagnose | Previous treatment | Donor  No. | Preconditioning therapy | | | | Dose M/kg | Response |
| --- | --- | --- | --- | --- | --- | --- | --- | --- | --- |
|  |  |  |  | FLU | CTX | VP-16 | TBI |  |  |
| P1 | DLBCL | RCHOP, REHAP, ASCT, Gemox | D1 | 25mg/m^2^*3 | 500mg/m^2^*2 | No | No | 1 | NR |
| P2 | DLBCL | RCHOP, REPOCH, Gemox | D2, D3 | 25mg/m^2^*3 | 500mg/m^2^*2 | No | No | 1.0, 4.1 | NR |
| P3 | DLBCL | RCHOP, RDHAP | D2 | 25mg/m^2^*3 | 500mg/m^2^*2 | No | No | 4.1 | NR |
| P4 | B-ALL | VDCLP, CAM, Hyper CVAD AB | D2 | 25mg/m^2^*3 | 500mg/m^2^*2 | No | No | 4.6 | NR |
| P5 | B-ALL | VDCP, CAM, Hyper CVAD AB, CAT, VDCP+E, | D4 | 25mg/m^2^*3 | 500mg/m^2^*2 | No | No | 5 | NR |
| P6 | B-ALL | VDLP, CAT, MEA, VICLP, CAT, VICD, EA, FLAG | D5 | 25mg/m^2^*3 | 500mg/m^2^*2 | No | No | 5 | NR |
| P7 | B-ALL | VDCLP, CAM, Hyper CVAD AB, EAD, VDCLP+VP-16 | D5 | 25mg/m^2^*3 | 500mg/m^2^*2 | 100mg*2 | 2Gy×3 | 5.4, 6.0 | CRi |
| P8 | B-ALL | VDCLP, CAM, VDP, Hyper CVAD AB | D6 | 25mg/m^2^*3 | 500mg/m^2^*2 | 100mg*2 | 2Gy×3 | 4.4 | CRi |
| P9 | B-ALL | VDCP+P, Hyper CVAD AB | D7 | 25mg/m^2^*3 | 500mg/m^2^*2 | 100mg*2 | 2Gy×3 | 5.6 | CRi |
