# Supplementary material for: Allogeneic CAR-T cells with of HLA-A/B and TRAC disruption exhibit promising antitumor capacity against B cell malignancies
Source: Cancer Immunol Immunother. 2024 Jan 17;73(1):13. doi: 10.1007/s00262-023-03586-1 (PMC10794471; doi:10.1007/s00262-023-03586-1)
Supplement: Supplementary file 6 — Supplementary file6 (DOC 33 KB) [file 262_2023_3586_MOESM6_ESM.doc]

**Supplementary Table 2 HLA types of patients and donors**

|  | P6 | P7 | P8 | P9 | D4 | D5 | D6 | D7 |
| --- | --- | --- | --- | --- | --- | --- | --- | --- |
| HLA-A* | 11:01,26:01 | 02:01,  11:01 | 02:09,26:01 | 02:06,26:01 | 02:06,11:01 | 02:03,11:02 | 30:01,30:01 | 02:01,24:02 |
| HLA-B* | 08:01,51:01 | 40:01,  40:01 | 07:02,37:01 | 35:01,35:03 | 15:25,39:01 | 38:02,39:01 | 13:02,13:02 | 15:11,  40:02 |
| HLA-C* | 07:02,14:02 | 03:04,  07:02 | 06:02,16:02 | 03:03,12:03 | 04:03,07:02 | 07:02,07:02 | 06:02,06:02 | 03:03,03:03 |
| HLA-DRB1* | 03:01,09:01 | 08:03,  15:01 | 01:01,11:04 | 13:01,14:03 | 08:03,12:02 | 08:03,16:02 | 07:01,07:01 | 15:01,15:01 |
| HLA-DPB1* | N/A | N/A | N/A | N/A | 02:01,19:01 | 02:02,05:01 | N/A | 02:01,05:01 |
| HLA-DQB1* | 02:01,03:03 | 06:01,  06:02 | 03:01;05:01 | 03:01,06:03 | N/A | N/A | 02:02,02:02 | N/A |
